# Supplementary material for: Splice-Junction-Based Mapping of Alternative Isoforms in the Human Proteome
Source: Cell Rep. Author manuscript; Available in PMC 2020 Jan 15. (PMC6961840; doi:10.1016/j.celrep.2019.11.026)
Supplement: 2 [file NIHMS1546469-supplement-2.zip › DF1/PXD006675/RightVentricle/RightVentricle_28_NEBL_TTQQNISAVK.pdf]

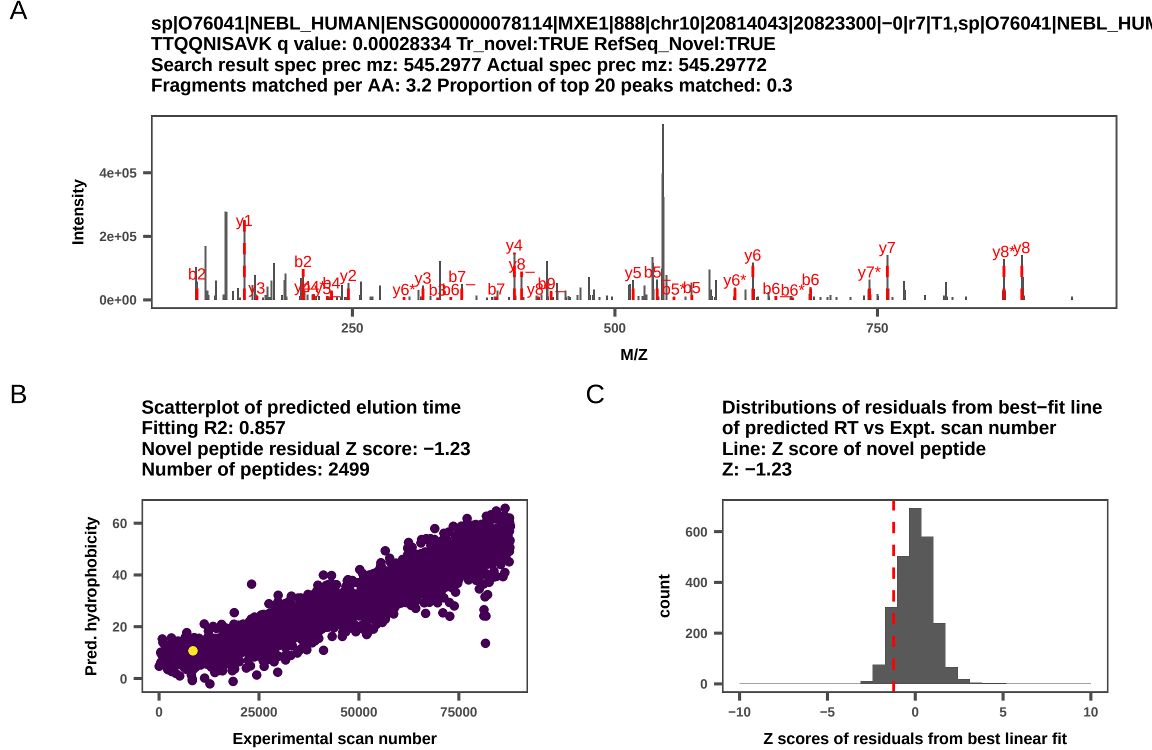
A
sp|O76041|NEBL_HUMAN|ENSG00000078114|MXE1|888|chr10|20814043|20823300|−0|r7|T1,sp|O76041|NEBL_HUM
TTQQNISAVK q value: 0.00028334 Tr_novel:TRUE RefSeq_Novel:TRUE
Search result spec prec mz: 545.2977 Actual spec prec mz: 545.29772
Fragments matched per AA: 3.2 Proportion of top 20 peaks matched: 0.3
Intensity
4e+05
2e+05
0e+00
y1
b2
y3
b2
y2
y3
b7
b7
y4
y8
y5
b5
y6
b6
y7
y7*
y8*
y8
250
500
750
M/Z
B
Scatterplot of predicted elution time
Fitting R2: 0.857
Novel peptide residual Z score: −1.23
Number of peptides: 2499
Pred. hydrophobicity
60
40
20
0
0
25000
50000
75000
Experimental scan number
C
Distributions of residuals from best−fit line
of predicted RT vs Expt. scan number
Line: Z score of novel peptide
Z: −1.23
count
600
400
200
0
−10
−5
0
5
10
Z scores of residuals from best linear fit
